# What is the mechanism of formation of hydroxyaluminosilicates?

**DOI:** 10.1038/srep30913

**Published:** 2016-08-01

**Authors:** James Beardmore, Xabier Lopez, Jon I. Mujika, Christopher Exley

**Affiliations:** 1 Birchall Centre, Lennard-Jones Laboratories, Keele University, Staffordshire, ST5 5BG, UK; 2 Kimika Fakultatea, Euskal Herriko Unibertsitatea UPV/EHU, and Donostia International Physics Center (DIPC), P.K. 1072, 20080 Donostia, Euskadi, Spain

## Abstract

The formation of hydroxyaluminosilicates is integral to the biogeochemical cycles of aluminium and silicon. The unique inorganic chemistry which underlies their formation explains the non-essentiality in biota of both of these elements. However, the first steps in the formation of hydroxyaluminosilicates were hitherto only theoretical and plausibly only accessible *in silico*. Herein we have used computational chemistry to identify and define for the first time these unique and ultimately critically important reaction steps. We have used density-functional theory combined with solvent continuum models to confirm first, the nature of the reactants, an aluminium hydroxide dimer and silicic acid, second, the reaction products, two distinct hydroxyaluminosilicates A and B and finally, how these are the precursors to highly insoluble hydroxyaluminosilicates the role of which has been and continues to be to keep inimical aluminium out of biota.

Silicon and aluminium are the second and third most abundant elements of the Earth’s crust^1^. Paradoxically neither element is essential to life and aluminium is inimical to biota. Silicon’s non-essentiality is reflected both in its non-existent biochemistry, there are no known Si-C, Si-N, Si-O-C…etc. bonds in biota, and its limited inorganic chemistry under physiological conditions^2^. The latter only consists of (i) the autocondensation of silicic acid (Si(OH)_4_) in forming silica (nSiO_2_•mH_2_O), (ii) the reaction of Si(OH)_4_ with ammonium molybdate to form molybdosilicic acid complexes (the basis for the spectrophotometric determination of Si(OH)_4_) and (iii) the reaction of Si(OH)_4_ with aluminium hydroxide (Al(OH)_3(s)_) to form hydroxyaluminosilicates (HAS). The formation of HAS is suggested as a basis for the essentiality of silicon in keeping aluminium out of biota^3^,^4^. Therefore the unique inorganic chemistry of the formation of HAS has been critical in the non-selection of aluminium in biochemical evolution and it continues to be important in combating the ecotoxicity of aluminium including human exposure to aluminium^5^.

The mechanism of formation of HAS proceeds via the competitive condensation of Si(OH)_4_ across hydroxyl groups on a template of Al(OH)_3(s)_^6^. The reaction is competitive because Si(OH)_4_ competes with the further hydroxylation or autocondensation of Al(OH)_3(s)_ and its eventual precipitation as gibbsite^6^,^7^. Two discrete forms of HAS, known as HAS_A_ and HAS_B_, have been identified^7^. HAS_A_ is the predominant HAS formed in solutions in which the initial concentration of Si(OH)_4_ is less than or equal to the total aluminium while HAS_B_ predominates where the initial concentration of Si(OH)_4_ is at least twice that of aluminium. Al(OH)_3(s)_ is a prerequisite to the formation of HAS_A_ which is itself required for the formation of HAS_B_. Filterable solids of HAS_A_ have an elemental Si:Al ratio of 0.50 and their structure is dominated by Si coordinated through three Si-O-Al linkages (Q^3^3Al) to Al(III) in octahedral geometry (Al^VI^). The solid phase of HAS_B_ has an elemental Si:Al ratio of 1.0 and Al(III) is found in tetrahedral (Al^IV^) and octahedral (Al^VI^) geometries in equal amounts^7^. This shift from octahedral to tetrahedral geometry is supported by changes in Si coordination from Q^3^(3Al), the signature for HAS_A_, to Q^3^(1-2Al)^7^. Atomic force microscopy was used to show that HAS solids adopt discrete morphologies with HAS_A_ forming flat (1–2 nm) rectangular (up to 170 nm in length) particles while HAS_B_ are also flat (1–2 nm) but discoid (up to 40 nm in diameter) particles^8^.

Present understanding of the formation and structures of HAS_A_ and HAS_B_ has, in the main, been obtained from solid state measurements of air-dried, filterable solids^9^. These data have allowed the formulation of potential reaction pathways beginning with monomeric and soluble reaction moieties, Al^3+^_(aq)_ and Si(OH)_4_, and finishing with precipitates of HAS_A_ and HAS_B_. The intermediates in this series of reactions are potentially myriad and their identities are certainly inaccessible through conventional bench-top analytical chemistry. Herein we have, for the first time, applied density-functional methods of computational chemistry to elucidate reaction intermediates in the formation of HAS_A_ and HAS_B_ and thereby support current solution and solid state data on these critical phases in the biogeochemistry of both aluminium^1^ and silicon^2^.

## Methods

### Density functional theory calculations

All geometrical optimisations were carried out in aqueous-phase using the Gaussian 09 suite of programmes^10^, B3LYP functional^11^,^12^,^13^,^14^ and the 6–31++G(d,p) basis set^15^,^16^. The B3LYP functional has previously been shown to be effective for modelling interactions involving aluminium^17^. To confirm that optimised structures were real minima on the potential energy surfaces, frequency calculations were carried out at the same level of theory. All structures showed positive force constants for all the normal modes of vibration. The frequencies were then used to evaluate the zero-point vibrational energy (ZPVE) and the thermal (*T* = 298 K) vibrational corrections to the enthalpies and Gibbs free energies within the harmonic oscillator approximation. To calculate the entropy, the different contributions to the partition function were evaluated using the standard statistical mechanics expressions in the canonical ensemble and the harmonic oscillator and rigid rotor approximation. The solvent effect was introduced by using the self-consistent reaction field (SCRF) method with the polarised continuum model (PCM), using the integral equation formalism variant (IEFPCM)^18^. The electronic energies were refined by single-point energy calculations at the B3LYP/6-311++G(3df,2p) level of theory^19^,^20^. For the most important structures and to validate our results, we recalculated the energies with PBE0^21^,^22^ and M062-X^23^ functionals.

### Preparation of structures

**Figure 1:**
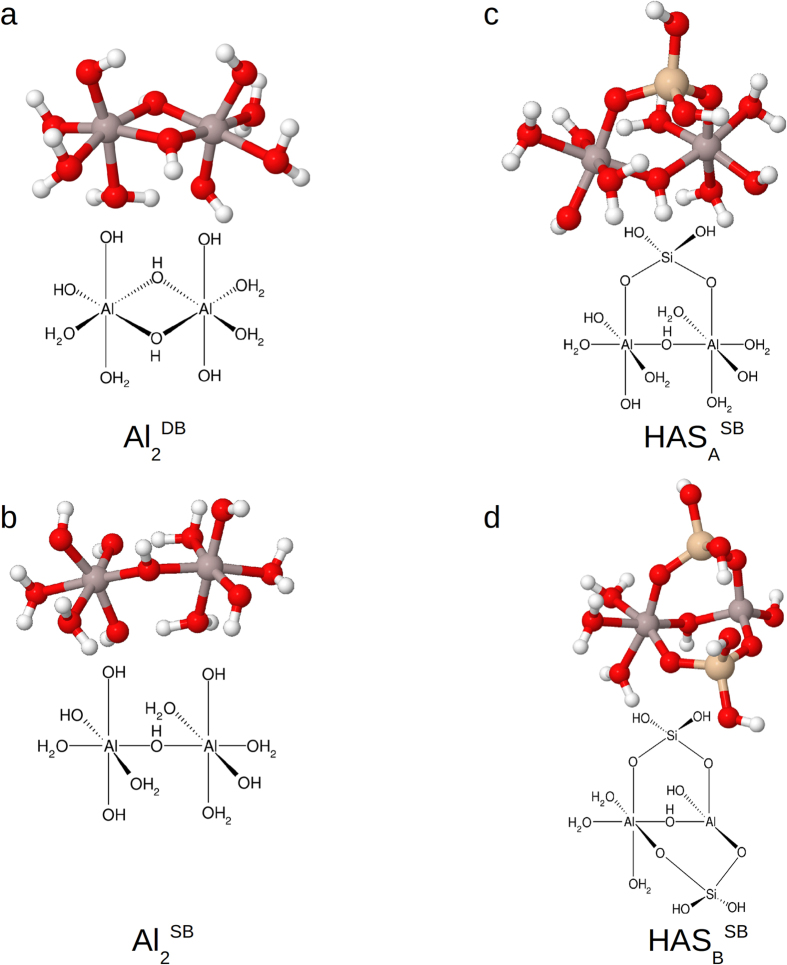
Example aluminium hydroxide and hydroxyaluminosilicate structures tested. (**a**) Double-bridge Al dimer, (**b**) Single-bridge Al dimer, (**c**) Single-bridge dimer b with Si(OH)_4_ bound at OH groups to form HAS_A_, (**d**) HAS_A_ structure comparable to c, with second Si(OH)_4_ bound to form HAS_B_, and 2H_2_O removed from the rightmost Al, leaving it with tetrahedral coordination.

**Figure 2:**
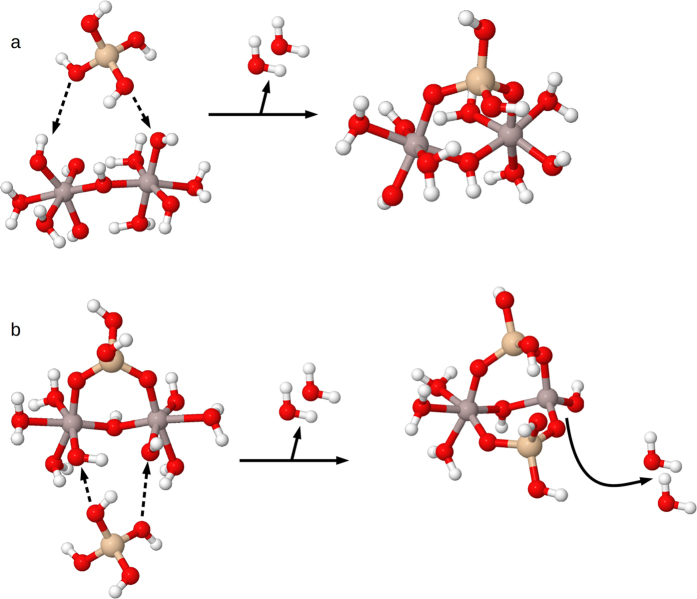
Binding of silicic acid molecule to (a) an aluminium hydroxide dimer to form HAS_A_, and (b) a HAS_A_ unit to form HAS_B_.

Based on the work of Bogatko and co-workers, aluminium hydroxide dimer templates (Al_2_ hereafter) were studied with aluminium atoms linked by double and single hydroxy bridges^17^ (e.g. structures a and b, Fig. 1), and at three charge states: dication, monocation and neutral. The first coordination shell was completed with water molecules and hydroxyl groups to produce the desired charge for each particular structure, with Al coordinated octahedrally. Several hydroxyaluminosilicate structures in both HAS_A_ and HAS_B_ forms (e.g. structures c and d, Fig. 1) were also investigated. Potential HAS_A_ particles in the same charge range were investigated by constructing an aluminium hydroxide dimer with either a double or single hydroxy bridge, and incorporating silicic acid (Si(OH)_4_) into the structure by using one coordination site on each Al atom to form an additional bridge between the two Al atoms, in the form of Al-O-Si-O-Al (structure a, Fig. 2; structure c, Fig. 1). The two remaining coordination sites on the incorporated Si were occupied with hydroxyl groups, as found on Si(OH)_4_ (structure a, Fig. 2; structure c, Fig. 1). Potential HAS_B_ structures were tested by incorporating a second Si into the structures, in the same manner (structure b, Fig. 2; structure d, Fig. 1).

Conformational changes occurred in some structures during geometry optimisation, for instance rearrangement of water and hydroxyl groups. This resulted in some conformations collapsing to identical ones already being investigated (*i.e.* some of the stable structures, as presented in the Supplementary material, could be reached from more than one different starting geometry). Some structures were found to be unstable, breaking apart during the geometry optimisation stage, releasing one or more water molecules from the structure.

**Figure 3:**
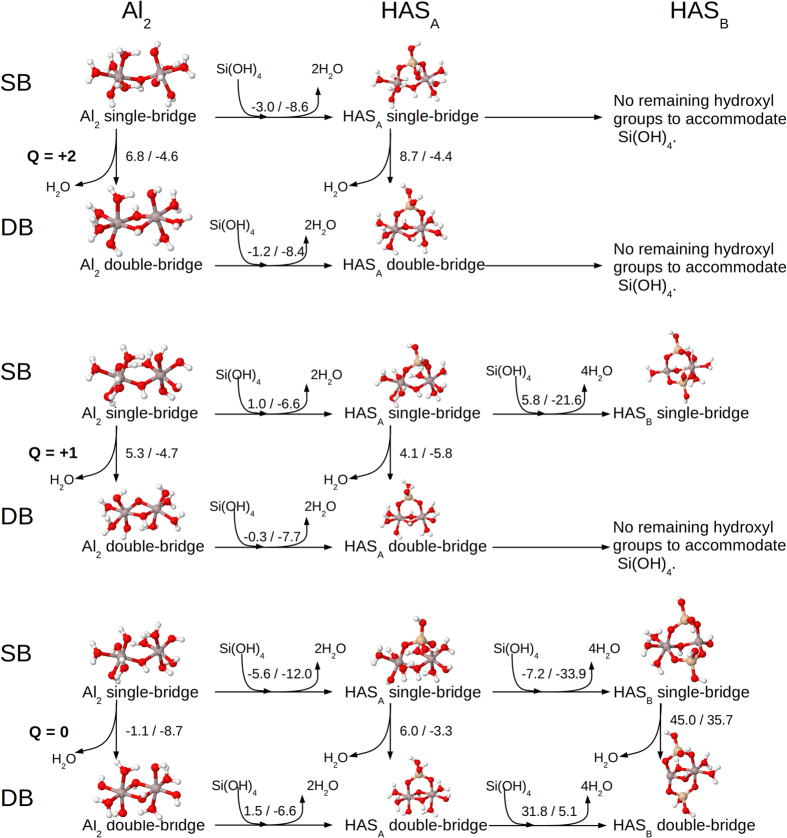
Enthalpies and free energy changes (ΔH_aq_/ΔG_aq_) for transitions between the most stable Al hydroxide dimer, HAS_A_ unit, and HAS_B_ unit of each charge and bridge-type. Units are in kcal/mol.

Geometries were also investigated where the aluminium atoms were linked by oxygen, rather than hydroxy bridges; however in almost all cases, geometry optimisation resulted in protons migrating from elsewhere to change the bridge to the hydroxy type. The exceptions to these were HAS_B_ and the AlSi monomer. As a result of this consistent conformational search, a set of 33 stable structures were obtained. For the sake of clarity, we only show the lowest-energy structures of each kind (Fig. 3), whereas all identified stable structures may be found in the Supplementary material (Tables S2–S6). The corresponding enthalpy (Δ*H*_*aq*_) and Gibbs free energy (Δ*G*_*aq*_) differences in the aqueous phase, for the formation of all these structures, can be found in several figures and tables in the Supplementary material: We show Δ*H*_*aq*_/Δ*G*_*aq*_ for the formation of single-bridge Al hydroxide dimer structures (Al_2_^SB^) in Table S2, and double-bridge ones (Al_2_^DB^) in Table S3; formation of HAS_A_ structures departing from aluminium hydroxide dimers in Tables S4 (single-bridge, HAS_A_^SB^) and S5 (double-bridge, HAS_A_^DB^), and finally, formation of HAS_B_ from HAS_A_ in Table S6.

### Condensation reactions

For the condensation reactions of Si(OH)_4_ on to Al_2_ hydroxide dimers to form HAS_A_ and subsequently HAS_B_, the condensation of a Si(OH)_4_ on to an Al_2_ or HAS_A_ unit was assumed to involve the interaction between two silanol groups on the Si(OH)_4_ and two suitably-distanced adjacent hydroxyl groups on the Al_2_ or HAS_A_. Each OH pair that interacts in this way must release one OH^−^ and one H^+^ into the bulk solution to form each Al-O-Si bond, *i.e.* the condensation of Si(OH)_4_ on to the structure releases two water molecules.

For the formation of HAS_A_, we consider


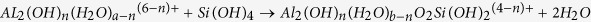


where *a* = 11 for single-bridge Al_2_ or *a* = 10 for double-bridge Al_2_; and *b* = 9 for single-bridge HAS_A_ or *b* = 8 for double-bridge HAS_A_.

For the formation of HAS_B_, we consider


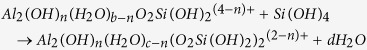


where *b* = 9 for single-bridge HAS_A_ or *b* = 8 for double-bridge HAS_A_; *c* is 7 for single-bridge HAS_B_ with octahedrally-coordinated Al, 6 for double-bridge HAS_B_ with octahedrally-coordinated Al, 5 for single-bridge HAS_B_ that has 50% tetrahedrally-coordinated Al, and 4 for double-bridge HAS_B_ that has 50% tetrahedrally-coordinated Al. *d* = 2 for HAS_B_ that has octahedrally-coordinated Al, and *d* = 4 for HAS_B_ that has 50% tetrahedrally-coordinated Al.

Thus, based on the experimental information^6^,^7^, we consider that the formation of each Al-O-Si bridge implies the release of a water molecule formed by the hydroxy group of the aluminium hydroxide and a proton of the incoming silanol group, as shown schematically in Fig. 2. Other condensation mechanisms, in which the silanol group would displace a water molecule from the first solvation layer of aluminium to release a H_3_O^+^, would alter the solution pH, a behaviour discarded based upon experimental evidence^6^.

## Results and Discussion

### Formation of Al_2_ dimer is more favourable than formation of AlSi monomer

First we analysed competition between the formation of aluminium hydroxide dimers and the condensation reaction between aluminium monomer and Si(OH)_4_ to form an AlSi monomer, as proposed by Browne and Driscoll^24^. Formation of an AlSi monomer required the release of one water molecule into the bulk solvent. We considered three aluminium monomers which differed in their charge states: Al(OH)(H_2_O)_5_^2+^, Al(OH)_2_(OH_2_)_4_^+^ and Al(OH)_3_(H_2_O)_3_. Both the condensation reaction of two aluminium monomer units to form an aluminium hydroxide dimer (Al_2_), and the condensation of aluminium hydroxide monomer and Si(OH)_4_ to form an AlSi hydroxide unit were exothermic and therefore possible in aqueous solution; however, the exothermicity of Al_2_ formation was much higher than the exothermicity of AlSi formation for any charge state. For example, the most favourable 1:1 interaction between an Al monomer and Si(OH)_4_ was between Al(OH)^2+^ and Si(OH)_4_ to form a AlSi^2+^ with Al bound to Si via an oxygen rather than hydroxy bridge. The enthalpy/entropy of reaction was −90.2/−84.6 kcal/mol. The most stable identified Al hydroxide dimer had a formation enthalpy/entropy of −199.1/−193.19 kcal/mol, when formed from two neutral Al(OH)_3_ monomers. The formation enthalpies of other Al dimers were of comparable values to the most favourable dimer (Table S1). Therefore, in solutions shown experimentally to result in HAS the formation of aluminium hydroxide dimers was always preferred to the reaction of Al monomer with Si(OH)_4_.

### HAS_A_ formation from Al_2_ hydroxide dimers is favourable, especially for neutral structures

Several stable aluminium hydroxide dimers were identified (Tables S2 and S3). We were also able to characterise several structures in which Si(OH)_4_ was incorporated into aluminium hydroxide dimers (Tables S4 and S5). Here we will only discuss the lowest-energy structures for each type (Fig. 3). There is a competitive situation between single-bridge and double-bridge aluminium dimer structures at the three different charge states. If we look at the enthalpies, both dicationic and monocationic Al_2_ show a preference for single-bridge structures, although when entropic corrections are introduced, double-bridge structures are preferred. For neutral structures, both enthalpic and free energies point to the double-bridge structure as the preferential form for Al_2_. However, the difference in Δ*H*_*aq*_ is only 1.1 kcal/mol at the B3LYP level of theory indicating that single-bridge structures are also accessible in aqueous solution. This is consistent with the results of Bogatko *et al*.^17^, who pointed to the coexistence of single-bridge and double-bridge Al_2_ structures in aqueous solution. Next, we analysed the reaction energy for the incorporation of Si(OH)_4_ into Al_2_ hydroxide dimers. The formation of HAS_A_ structures by the condensation of Si(OH)_4_ on to the Al dimer, and release of two water molecules, was favourable in several instances. Thus, according to Δ*G*_*aq*_ values, the formation of HAS_A_ structures was found to be an overall exothermic process in all cases. On the other hand, it was found that Δ*H*_*aq*_ was negative in four cases and only slightly positive (within the error of the calculation) in two cases: condensation of Si(OH)_4_ on to Al_2_^SB1+^ and neutral Al_2_^DB^. In general, therefore, there is a propensity to form HAS_A_ structures, with a preference for the addition of Si(OH)_4_ into neutral single-bridge Al_2_ hydroxide to form neutral single-bridge HAS_A_ structures, with values of −5.6/−12.0 kcal/mol for Δ*H*_*aq*_/Δ*G*_*aq*_.

### HAS_B_ formation from HAS_A_ is highly favourable from neutral single-bridge structures

For charged HAS_A_, taking into account the number of hydroxyl groups, there is only the possibility to condense a second Si(OH)_4_ on to an Al_2_^SB1+^ structure. When we do so, we observe a change in the coordination shell of one of the aluminium atoms to four-coordination and the release of two waters. Therefore, the overall reaction corresponds to the addition of a Si(OH)_4_ molecule, with the formation of two Al-O-Si bridges and release of four water molecules. The reaction Δ*H*_*aq*_ is positive with a value of 5.8 kcal/mol, however, the release of four water molecules increases the reaction entropy, and therefore, Δ*G*_*aq*_ is now quite negative, −21.6 kcal/mol. In the case of neutral HAS_A_, we were able to find HAS_B_ structures originating from both single-bridge and double-bridge HAS_A_ species. In both cases, one of the aluminium atoms changes its coordination from six to four, but only one case, the condensation of Si(OH)_4_ on to a neutral single-bridge HAS_A_ structure, is exothermic, with Δ*H*_*aq*_/Δ*G*_*aq*_ values of −7.2/−33.2 kcal/mol. Attempting to produce a HAS_B_ structure with Al linked via an oxygen atom, rather than a hydroxy bridge, by shifting the proton from the bridge to a hydroxyl group elsewhere on the structure, resulted in a Δ*H*_*aq*_/Δ*G*_*aq*_ energy penalty of 24.6/23.5 kcal/mol.

In all cases where HAS_B_ was formed, we observed a tendency for Si(OH)_4_ groups to orient adjacent to each other, rather than on opposite sides of the structure. However, neutral double-bridge HAS_B_ implied opposing Si(OH)_4_ groups, and this consistently lead to a structure with a high degree of strain and unfavourable energy. In summary, the formation of HAS_B_ via HAS_A_ is favourable from neutral single-bridge structures, leading to a dramatic change in the coordination shell of one Al atom (Fig. 3). We note that this is the exact behaviour that has been observed experimentally by NMR^7^.

### The formation of HAS_B_ is exothermic and requires specific orientation of hydroxyl groups

**Figure 4:**
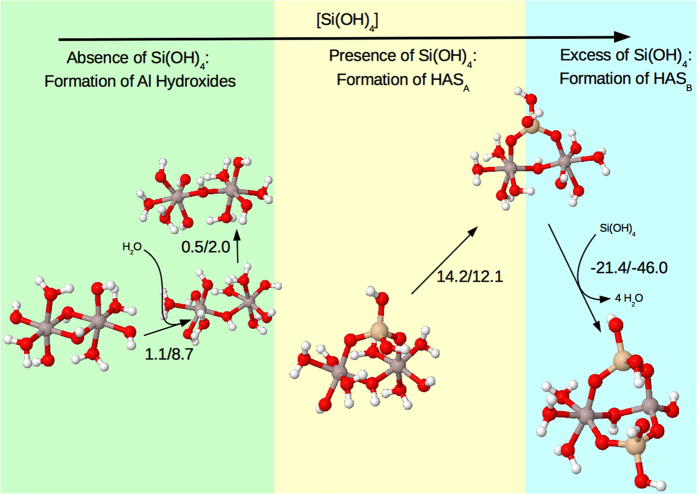
Most favourable reaction pathway for neutral structures as a function of Si(OH)_4_ concentration. Data extracted from Fig. 3, showing the most favourable Al hydroxide dimer, HAS_A_ and HAS_B_ structures, and the intermediates connecting these species. The figure shows schematically the influence of Si(OH)_4_ concentration in the formation of HAS_A_ and HAS_B_ species. Reaction-enthalpies and free energies in the aqueous phase are given in kcal/mol.

Experimental data^6^,^7^,^8^, and supported by the *in* silico measurements herein, suggest that neutral structures are the precursors to the formation of HAS (Fig. 4). Formation of aluminum hydroxide is a prerequisite for HAS formation. Our results for aluminum hydroxide dimer templates suggest that there is a competition between single-bridge and double-bridge structures in the case of charged species. Neutral Al hydroxides however tend to favour double-bridge species (although the energy differences are not as large as to prevent the formation of single-bridged structures at room-temperature) and are more favourable to aggregate. Repulsion between positively-charged molecules in solution may make the aggregation and growth of neutral Al hydroxide more competitive than that of positively-charged Al hydroxide, which would be consistent with existing experimental data. In order to form HAS_A_, a single-bridge Al hydroxide configuration is needed, structures that are easily accessible, implying small energy requirements. In addition, in order for Si(OH)_4_ to condense, the structure must shift its conformation, to provide two adjacent hydroxyl groups (spaced approximately 0.3 nm apart) for incorporation of Si(OH)_4_. This provides an additional Δ*G*/Δ*H* energy penalty of 0.5/2.0 kcal/mol (Fig. 4, Table 1). Thus, only moderate energy requirements were identified in the stages of HAS formation. The small barrier to HAS_A_ formation is consistent with laboratory observations that its formation is comparatively quick. The incorporation of Si(OH)_4_ into this structure with the release of two water molecules results in an enthalpy/entropic-change of −6.1/−14.0 kcal/mol, making the formation of HAS_A_ from Al hydroxide favourable over-all. To analyse the consistency of this theoretical picture, we have recalculated the energies of the structures of Fig. 4 and reevaluated the corresponding Δ*H*_*aq*_/Δ*G*_*aq*_ with two other functionals, PBE0 and M062-X (see Table 1), finding the same behaviour.

Laboratory observations point to relatively slow HAS_B_ formation and its high stability. Our results show that incorporation of a second Si(OH)_4_ into the structure requires a second pair of suitably-spaced hydroxyl groups, however, this requirement implies an important increase in energy, 14.2/12.1 kcal/mol. In addition, the incorporation of a second Si(OH)_4_ into the structure and release of four water molecules, results in a reaction energy Δ*H*_*aq*_/Δ*G*_*aq*_ of −21.4/−46.0 kcal/mol, making the formation of HAS_B_ from HAS_A_ very stable thermodynamically, and therefore, almost irreversible, in agreement with experimental data. It is important to note that the energy penalty for reorientation of the corresponding hydroxyl groups in HAS_A_ is much higher than for Al_2_ dimer structures, and is consistent with the experimentally known slow formation of HAS_B_ with respect to HAS_A_^7^. Notice that the spacing of the hydroxyl groups on the Si(OH)_4_ molecule is 0.3 nm, and the spacing remains constant when two of the groups participate in condensation on to the aluminium hydroxide dimer. The spacing of one out of each set of opposing functional groups on the aluminium hydroxide dimer is always approximately 0.3 nm. The opposing group may be spaced more widely occasionally, due to the angle formed at the hydroxyl group(s) bridging the aluminium atoms. On aluminium hydroxide dimers with two adjacent hydroxyl groups capable of accepting a single Si(OH)_4_, it is possible for these hydroxyl groups to be spaced slightly wider than 0.3 nm, however upon introduction of Si(OH)_4_ to the structure, the hydroxy bridge between the aluminium atoms rotates, altering the angle, thus reducing the gap to the favoured 0.3 nm. The slight angle at the hydroxy bridge between the aluminium atoms is also a likely factor in the second Si(OH)_4_ favouring hydroxyl groups that are adjacent (perpendicular) to the first Si(OH)_4_, rather than the opposing groups. Perpendicular silanol groups allow for less strain and torsion of the HAS_B_ molecule than opposing ones, due to the ability of the Al-OH-Al bridge to rotate to a position that allows both silanol groups to maintain their optimal 0.3 nm spacing. The favourability of single-bridge HAS structures over double-bridge variants may be in part due to the relative flexibility of a single hydroxy bridge compared to a double-bridge, with the single-bridge acting as a means of reducing strain across the rest of the structure. A more complete picture of the reaction mechanisms shown here would require calculation of corresponding transition states for all possible condensation reactions. Due to the high variety of structures calculated in this work, this task is beyond the scope of the present work and is the subject of future work. However, the agreement with experimental data supports our initial assumption that the formation of HAS_A_ and HAS_B_ can be understood in a simplified way by the thermodynamic requirements of the formation of ideal precursors for condensation of SiOH_4_ onto aluminium hydroxides (HAS_A_ formation) and HAS_A_ templates (HAS_B_ formation).

## Conclusions

The first, initial steps in the mechanism of formation of HAS have hitherto not been confirmed by experiment. One reason for this is the nature of the initial reactants. HAS are not true solution species which upon reaching saturation precipitate to give the now well characterised solid phases^7^. Herein we have confirmed previous suggestions that soluble aluminosilicates^24^ are formed in acidic solutions, specifically Al(H_2_O)_5_OSi(OH)_3_^2+^, and we have similarly been able to demonstrate that such soluble forms of aluminosilicates are not precursors to HAS as their formation is not favoured under conditions where Al(H_2_O)_3_(OH)_3(aq)_ spontaneously condenses to give Al(H_2_O)_3_(OH)_3(s)_. These are the conditions which support the formation of HAS and the simplest example of such is the reaction of a dimer of Al(H_2_O)_3_(OH)_3(s)_ with Si(OH)_4(aq)_, the reaction of a solute with a solid phase and not the reaction of two solutes to form a soluble HAS. There is no such thing as a soluble HAS. What is intriguing about this reaction is that a highly insoluble solid phase, amorphous aluminium hydroxide, is the prerequisite to the even more insoluble solid phases of HAS_A_ and HAS_B_. It was this novel form of non-solution chemistry which made the attribution of a solubility product to HAS_B_ a non-trivial task^25^. When two Al(H_2_O)_3_(OH)_3(s)_ react to form a dimer the Al atoms are initially linked by a single oxy/hydroxyl bridge which spontaneously rearranges to give the double oxy/hydroxyl bridge characteristic of aluminium hydroxide and eventually gibbsite. The answer as to which of these forms of the dimer was the precursor to HAS_A_ was answered previously in part by electron probe analyses^7^ of HAS_A_ which did not identify enough elemental oxygen to support a double-bridge in HAS_A_. This suggested that when an aluminium hydroxide dimer formed in the presence of Si(OH)_4_ the formation of HAS_A_ ‘stabilised’ the single oxy/hydroxyl bridge and such is fully supported by our computational analyses. The formation of HAS_A_ is competitive with respect to its precursor, an aluminium hydroxide dimer, and it will be the only reaction product where [Al_T_] in solution equals the [Si(OH)_4_] at which point half of the original [Si(OH)_4_] remains in solution effectively preventing the dissolution of HAS_A_. Evidence suggests that HAS_A_ can carry a slight positive charge, slowing its subsequent aggregation, and that remaining hydroxyl (Al-OH) linkages may be either opposite to or at right angles to Al-O-Si linkages. The latter is important because when the residual [Si(OH)_4_] exceeds the concentration of the HAS_A_ dimer by a factor of two or more (when the original [Si(OH)_4_] is more than twice the original [Al_T_]) HAS_A_ becomes the precursor for the formation of HAS_B_. The energy barrier for the condensation of a second molecule of Si(OH)_4_ coming from its significant excess in solution. The subsequent high stability of HAS_B_ being then achieved by the spontaneous loss of two water molecules and a shift in the geometry of one of the aluminium atoms from octahedral to tetrahedral. This is equivalent to a room temperature dehydroxylation reaction and as we have shown herein its success depends upon the availability of hydroxyl groups on HAS_A_ which are approximately 0.3 nm apart and at right angles to the Al-O-Si linkages. The dehydroxylation and subsequent shift in geometry also ensures neutrality of the resultant HAS_B_ and its rapid subsequent aggregation towards filterable solids.

The reactions described herein and confirmed for the first time by computational methods have wider significance than simply their unique inorganic chemistry. These reactions are fundamental to the geochemical cycling of aluminium^1^ and integral to the biological availability of the non-essential but potentially toxic free metal cation, Al^3+^_(aq)_. In particular humans are experiencing a burgeoning exposure to aluminium in everyday life^26^ and such is implicated in a number of chronic diseases including neurological conditions such as Alzheimer’s disease^27^. The formation of HAS has been shown to protect against the toxicity of aluminium^3^,^28^ and is believed to underlie the mechanism whereby silicon-rich mineral waters facilitate the removal of aluminium from the human body^25^. The therapeutic potential of Si(OH)_4_ was recently reviewed^9^ but it was Louis Pasteur (June 13^th^ 1878) who is attributed as saying that *“effects of silicic acid are destined to play a great and major role in therapy”* and the formation of HAS may well be the mechanism.

## Additional Information

**How to cite this article**: Beardmore, J. *et al*. What is the mechanism of formation of hydroxyaluminosilicates? *Sci. Rep.* **6**, 30913; doi: 10.1038/srep30913 (2016).

## Supplementary Material

Supplementary Information

## Figures and Tables

**Table 1:** Enthalpy and Free Energy changes for each stage of the reaction pathway in kcal/mol depicted in Fig. 4, calculated with three different functionals: B3LYP, PBE0 and M062X.

Reaction	B3LYP		PBE0		M062X	
	ΔH	ΔG	ΔH	ΔG	ΔH	ΔG
*Al*_2_^*DB*^ + *H*_2_*O* → *Al*_2_^*SB*^	1.1	8.7	0.6	10.3	0.6	9.3
*Al*_2_^*SB*^ → *Al*_2_^*SB*’^	0.5	2.0	1.0	0.6	0.4	0.4
*Al*_2_^*SB*’^ + *Si*(*OH*)_4_ → *HAS*_*A*_^*SB*^ + 2*H*_2_*O*	−6.1	−14.0	−6.7	−14.9	−8.2	−15.4
*HAS*_*A*_^*SB*^ → *HAS*_*A*_^*SB*’^	14.2	12.1	15.7	13.7	17.1	15.9
*HAS*_*A*_^*SB*’^ + *Si*(*OH*)_4_ → *HAS*_*B*_^*SB*^ + 4*H*_2_*O*	−21.4	−46.0	−16.3	−43.6	−11.1	−38.5

## References

[b1] Exley C. A biogeochemical cycle for aluminium? J. Inorg. Biochem. 97, 1–7 (2003). 14507454 10.1016/s0162-0134(03)00274-5

[b2] Exley C. Silicon in life: A bioinorganic solution to bioorganic essentiality. J. Inorg. Biochem. 69, 139–144 (1998).

[b3] Birchall J. D., Exley C., Chappell J. S. & Phillips M. J. Acute toxicity of aluminium to fish eliminated in silicon-rich acid waters. Nature 338, 146–148 (1989).

[b4] Birchall J. D. The role of silicon in biology. Chem. Brit. 26, 141–144 (1990).

[b5] Exley C. Darwin, natural selection and the biological essentiality of aluminium and silicon. Trends. Biochem. Sci. 34, 589–593 (2009). 19773172 10.1016/j.tibs.2009.07.006

[b6] Exley C. & Birchall J. D. A mechanism of hydroxyaluminosilicate formation. Polyhedron 12, 1007–1017 (1993).

[b7] Doucet F. J. . The formation of hydroxyaluminosilicates of geochemical and biological significance. Geochim. Cosmochim. Acta 65, 2461–2467 (2001).

[b8] Doucet F. J., Rotov M. E. & Exley C. Direct and indirect identification of the formation of hydroxyaluminosilicates in acidic solutions. J. Inorg. Biochem. 87, 71–79 (2001). 11709216 10.1016/s0162-0134(01)00317-8

[b9] Exley C. Reflections upon and recent insight into the mechanism of formation of hydroxyaluminosilicates and the therapeutic potential of silicic acid. Coord. Chem. Rev. 256, 82–88 (2012).

[b10] Frisch M. J. . Gaussian 09 Revision A.1. Gaussian Inc., Wallingford CT (2009).

[b11] Becke A. D. Density-functional thermochemistry. 3. The role of exact exchange. J. Chem. Phys. 98, 5648–5652 (1993).

[b12] Becke A. D. Density-functional exchange-energy approximation with correct asymptotic-behavior. Phys. Rev. A 38, 3098–3100 (1988). 10.1103/physreva.38.3098 9900728

[b13] Lee C., Yang W. & Parr R. Development of the collesalvetti correlation-energy formula into a functional of the electron density. Phys. Rev. B 37, 785–789 (1988). 10.1103/physrevb.37.785 9944570

[b14] Vosko S. H., Wilk L. & Nusair M. Accurate spin-dependent electron liquid correlation energies for local spin-density calculations - a critical analysis. Can. J. Phys. 58, 1200–1211 (1980).

[b15] Francl M. M. . Self‐consistent molecular orbital methods. XXIII. A polarization‐type basis set for second‐row elements. J. Chem. Phys. 77, 3654–3665 (1982).

[b16] Ditchfield R., Hehre W. J. & Pople J. A. Self‐consistent molecular‐orbital methods. IX. An extended Gaussian‐type basis for molecular‐orbital studies of organic molecules. J. Chem. Phys. 54, 724–728 (1971).

[b17] Bogatko S. & Geerlings P. Factors influencing Al^3+^-dimer speciation and stability from density functional theory calculations. Phys. Chem. Chem. Phys. 14, 8058–8066 (2012). 22569929 10.1039/c2cp40885f

[b18] Tomasi J., Mennucci B. & Cammi R. Quantum mechanical continuum solvation models. Chem. Rev. 105, 2999–3093 (2005). 16092826 10.1021/cr9904009

[b19] Krishnan R., Binkley J. S., Seeger R. & Pople J. A. Self‐consistent molecular orbital methods. XX. A basis set for correlated wave functions. J. Chem. Phys. 72, 650–654 (1980).

[b20] McLean A. D. & Chandler G. S. Contracted Gaussian basis sets for molecular calculations. I. Second row atoms, Z = 11–18. J. Chem. Phys. 72, 5639–5648 (1980).

[b21] Perdew J. P., Burke K. & Ernzerhof M. Generalized gradient approximation made simple. Phys. Rev. Lett. 77, 3865–3868 (1996). 10062328 10.1103/PhysRevLett.77.3865

[b22] Perdew J. P., Burke K. & Ernzerhof M. Errata: Generalized gradient approximation made simple [phys. rev. lett. 77, 3865 (1996)]. Phys. Rev. Lett. 78, 1396 (1997). 10.1103/PhysRevLett.77.3865 10062328

[b23] Zhao Y. & Truhlar D. G. The M06 suite of density functionals for main group thermochemistry, thermochemical kinetics, noncovalent interactions, excited states, and transition elements: Two new functionals and systematic testing of four M06-class functionals and 12 other functionals. Theor. Chem. Acc. 120, 215–241 (2007).

[b24] Browne B. A. & Driscoll C. T. Soluble aluminum silicates: Stoichiometry, stability, and implications for environmental geochemistry. Science 256, 1667–1670 (1992). 17841088 10.1126/science.256.5064.1667

[b25] Schneider C., Doucet F., Strekopytov S. & Exley C. The solubility of an hydroxy-aluminosilicate. Polyhedron 23, 3185–3191 (2004).

[b26] Exley C. Human exposure to aluminium. Environ. Sci. Proc. Impact. 15, 1807–1816 (2013). 10.1039/c3em00374d 23982047

[b27] Exley C. What is the risk of aluminium as a neurotoxin? Expert Rev. Neurother. 14, 589–591 (2014). 24779346 10.1586/14737175.2014.915745

[b28] Davenward S. . Silicon-rich mineral water as a non-invasive test of the ‘aluminium hypothesis’ in Alzheimer’s disease. J. Alzheimers. Dis. 33, 423–430 (2013). 22976072 10.3233/JAD-2012-121231

