# Supplementary material for: What is the mechanism of formation of hydroxyaluminosilicates?
Source: Sci Rep. 2016 Aug 1;6:30913. doi: 10.1038/srep30913 (PMC4967899; doi:10.1038/srep30913)
Supplement: Supplementary Information [file srep30913-s1.pdf]

What is the mechanism of formation of hydroxyaluminosilicates?

James Beardmore[1] Xabier Lopez[2] Jon I. Mujika[2] Christopher Exley[1]

[1]Birchall Centre, Lennard-Jones Laboratories, Keele University, Staffordshire, ST5 5BG, UK.
[2]Kimika Fakultatea, Euskal Herriko Unibertsitatea UPV/EHU, and Donostia International Physics Center (DIPC), P.K. 1072, 20080 Donostia, Euskadi, Spain.

Supplementary Information

Table S1 - Reaction energies for formation of AlSi monomer and Al_2 dimers.

Reaction	ΔH_{aq} kcal/Mol	ΔG_{aq} kcal/Mol
$Al(OH)(H_2O)_5^{2+} + Si(OH)_4 \rightarrow Al(H_2O)_5OSi(OH)_3^{2+} + H_2O$	-90.19	-84.61
$Al(OH)_2(H_2O)_4^{1+} + Si(OH)_4 \rightarrow Al(H_2O)_4(OH)OSi(OH)_3^{1+} + H_2O$	-89.38	-84.47
$Al(OH)_3(H_2O)_3 + Si(OH)_4 \rightarrow Al(H_2O)_3(OH)_2OSi(OH)_3 + H_2O$	-88.84	-84.91
$2\,Al(OH)(H_2O)_5^{2+} \rightarrow Al_2(OH)_4(H_2O)_7^{2+} + H_3O^+ + H^+$	-197.92	-180.04
$2\,Al(OH)_2(H_2O)_4^{+} \rightarrow Al_2(OH)_5(H_2O)_6^{+} + H_3O^+$	-172.93	-166.53
$2\,Al(OH)_3(H_2O)_3 \rightarrow Al_2(OH)_6(H_2O)_5 + H_2O$	-199.14	-193.19

Identified Stable Structures

Next we provide a summary of all stable structures found for Al hydroxide dimer, HAS_A and HAS_B templates. As said in the manuscript, we performed a consistent DFT conformational search departing from various type of structures. In some of them, during geometry optimisation, there was an important rearrangement of water and hydroxyl groups, resulting in some conformations collapsing to identical ones already being investigated. In addition, Some structures were found to be unstable, breaking apart during the geometry optimisation stage, releasing one or more water molecules from the structure. One should notice in this respect that the PCM formalism used in the calculations to approximate the bulk solvent has limitations. While it is a good approximation of the "average" effects of a bulk solvent, it has shortcoming to takes into account any possible interactions of specific individual water molecules in the direct vicinity of the structure to be optimised. When structures broke apart during the optimisation stage of calculations, the addition of explicit water molecules into the simulation, outside the first coordination shell, might have rendered some of them stable, "guarding" or "shielding" parts of the structure from others. This still may not change

the results qualitatively, because fragmentation of the structure implies strain and torsion, which implies a higher energy state than similar structures that have already been identified as stable.

Table S2 - Single-bridge Al_2 dimers. The relative free-energy and enthalpy differences between each isomer and the most favourable isomer are given in kcal/mol in the form ($\Delta H_{aq} / \Delta G_{aq}$).

$[Al_2^{SB}]^{2+}$	$[Al_2^{SB}]^{1+}$	$[Al_2^{SB}]^{0}$
	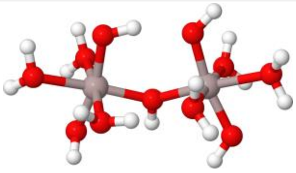 (234.5/232.2)	
	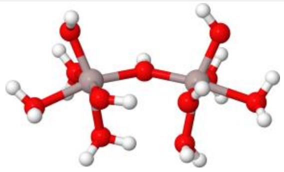 (20.7/19.6)	
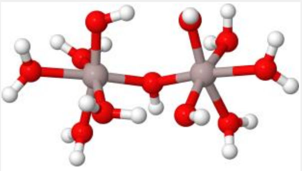 (11.0/10.1)	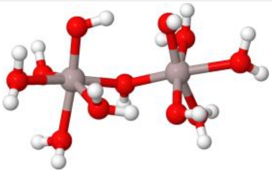 (14.4/13.1)	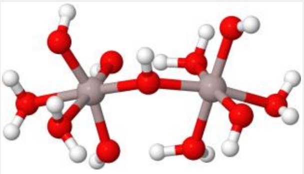 (0.5/2.0)
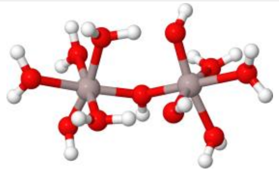 (0.0/0.0)	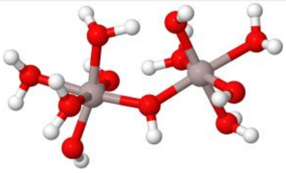 (0.0/0.0)	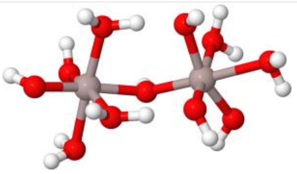 (0.0/0.0)

Table S3 - Double-bridge Al_2 dimers. The relative free-energy and enthalpy differences between each isomer and the most favourable isomer are given in kcal/mol in the form ($\Delta H_{aq} / \Delta G_{aq}$).

$[Al_2^{DB}]^{2+}$	$[Al_2^{DB}]^{1+}$	$[Al_2^{DB}]^{0}$
		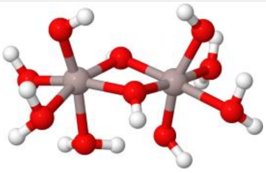 (159.3/157.7)
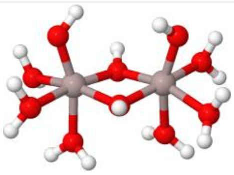 (7.8/6.6)	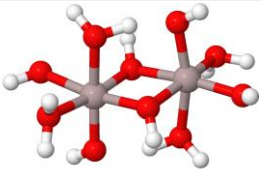 (199.3/201.3)	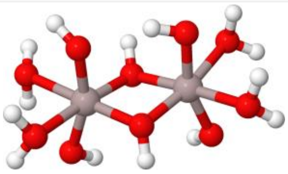 (13.6/10.7)
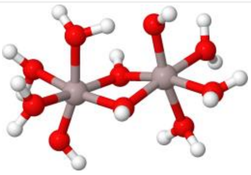 (0.0/0.0)	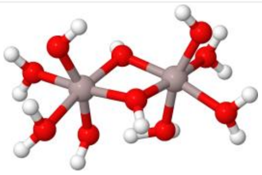 (0.0/0.0)	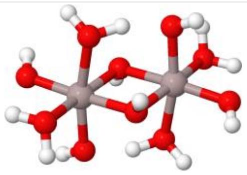 (0.0/0.0)

Table S4 - Single-bridge HAS_A units. The relative free-energy and enthalpy differences between each isomer and the most favourable isomer are given in kcal/mol in the form ($\Delta H_{aq} / \Delta G_{aq}$).

$[HAS_A{}^{SB}]^{2+}$	$[HAS_A{}^{SB}]^{1+}$	$[HAS_A{}^{SB}]^{0}$
		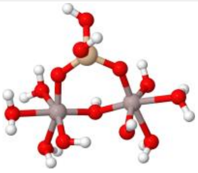 (14.2/12.1)
	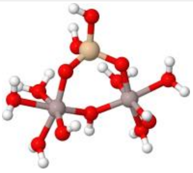 (5.7/5.7)	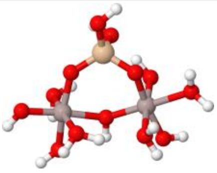 (12.6/13.5)
	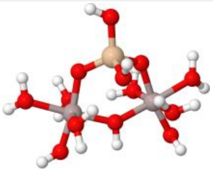 (1.1/2.8)	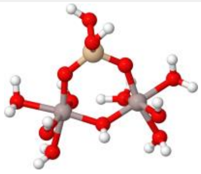 (2.4/1.4)
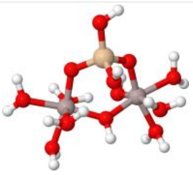 (0.0/0.0)	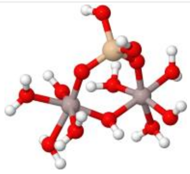 (0.0/0.0)	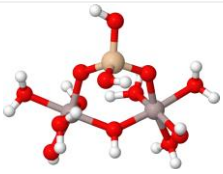 (0.0/0.0)

Table S5 - Double-bridge HAS_A units. The relative free-energy and enthalpy differences between each isomer and the most favourable isomer are given in kcal/mol in the form ($\Delta H_{aq} / \Delta G_{aq}$).

$[HAS_A^{DB}]^{2+}$	$[HAS_A^{DB}]^{1+}$	$[HAS_A^{DB}]^{0}$
		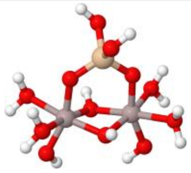 (0.7/-1.8)
	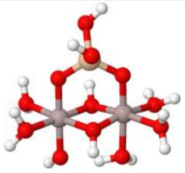 (1.4/2.8)	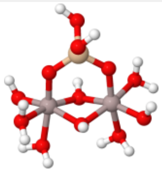 (0.1/-1.4)
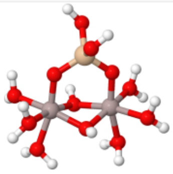 (0.0/0.0)	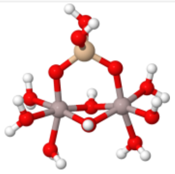 (0.0/0.0)	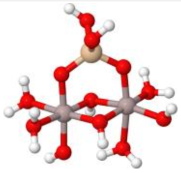 (0.0/0.0)

Table S6 - HAS_Bunits. The relative free-energy and enthalpy differences between each isomer and the most favourable isomer are given in kcal/mol in the form ($\Delta H_{aq} / \Delta G_{aq}$).

$[HAS_B^{SB}]^{1+}$	$[HAS_B^{SB}]^{0}$	$[HAS_B^{DB}]^{0}$
	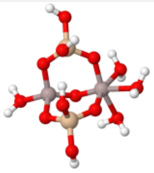 (24.6/23.5)	
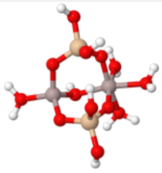 (0.0/0.0)	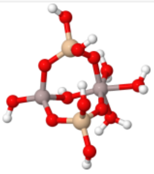 (0.0/0.0)	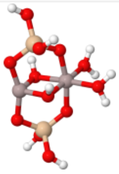 (0.0/0.0)

Absolute Energies of Identified Stable Structures

Table S7 - Absolute energies of most optimal identified structures

Structure	H_{aq} a.u.	G_{aq} a.u.
$[Al_2^{SB}]^{2+}$	-1323.540804	-1323.614082
$[Al_2^{SB}]^{1+}$	-1323.112015	-1323.186573
$[Al_2^{SB}]^{0}$	-1322.670700	-1322.746336
$[Al_2^{DB}]^{2+}$	-1247.084804	-1247.154783
$[Al_2^{DB}]^{1+}$	-1246.658390	-1246.727528
$[Al_2^{DB}]^{0}$	-1246.227305	-1246.293639
$[HAS_A^{SB}]^{2+}$	-1763.777088	-1763.855132
$[HAS_A^{SB}]^{1+}$	-1763.341919	-1763.424553
$[HAS_A^{SB}]^{0}$	-1762.911054	-1762.992915
$[HAS_A^{DB}]^{2+}$	-1687.318154	-1687.395501
$[HAS_A^{DB}]^{1+}$	-1686.890335	-1686.967187
$[HAS_A^{DB}]^{0}$	-1686.456358	-1686.531597
$[HAS_B^{SB}]^{1+}$	-2050.673966	-2050.753157
$[HAS_B^{SB}]^{0}$	-2050.263776	-2050.341192
$[HAS_B^{DB}]^{0}$	-1973.746984	-1973.817772
